# Cannabis, a Significant Risk Factor for Violent Behavior in the Early Phase Psychosis. Two Patterns of Interaction of Factors Increase the Risk of Violent Behavior: Cannabis Use Disorder and Impulsivity; Cannabis Use Disorder, Lack of Insight and Treatment Adherence

**DOI:** 10.3389/fpsyt.2018.00294

**Published:** 2018-07-04

**Authors:** Valerie Moulin, Philipp Baumann, Mehdi Gholamrezaee, Luis Alameda, Julie Palix, Jacques Gasser, Philippe Conus

**Affiliations:** ^1^Unit for Research in Legal Psychiatry and Psychology, Department of Psychiatry, Institute of Forensic Psychiatry, Centre Hospitalier Universitaire Vaudois, Lausanne University Hospital, Lausanne, Switzerland; ^2^Service of General Psychiatry, Department of Psychiatry, Centre Hospitalier Universitaire Vaudois, Lausanne, Switzerland; ^3^Department of Psychiatry, Center for Psychiatric Epidemiology and Psychopathology, Centre Hospitalier Universitaire Vaudois, Lausanne, Switzerland; ^4^Department of Psychosis Studies, Institute of Psychiatry, Psychology and Neuroscience, King's College London, London, United Kingdom; ^5^Unit for Research in Schizophrenia, Departement of Psychiatry, Center for Psychiatric Neuroscience and Service of General Psychiatry, Centre Hospitalier Universitaire Vaudois, Lausanne University Hospital, Lausanne, Switzerland

**Keywords:** cannabis use disorder, violent behavior, impulsivity, insight, early phase of psychosis, profiles

## Abstract

**Background:** Previous literature suggests that prevalence of cannabis use in the early phase of psychosis is high, and that early psychosis patients are at high-risk for violent behavior. However, the link between cannabis use and violent behavior in early psychosis patients is unclear. We carried out a study on a sample of early psychosis patients, in order to explore the impact of cannabis use on the risk of violent behavior (VB), while taking into account (1) potential confounding factors and, (2) interactions with other dynamic risk factors of VB.

**Method:** In a sample of 265 early psychosis patients, treated at the Treatment and Early Intervention in Psychosis Program (TIPP) in Lausanne, we used logistic regression models to explore the link between various dynamic risk factors of VB [positive symptoms, substance use disorder (drugs including cannabis, alcohol and others drugs), insight, impulsivity, affective instability, and treatment adherence], and VB occurring during treatment. In order to understand hierarchical effects attributable to the combinations of risk factors on VB we conducted a Classification and Regression Tree (CART).

**Results:** Our results show that cannabis use disorder is a risk factor for VB. The associations among risk factors suggest the presence of two patient profiles with an increased rate of VB: the first is composed of patients with cannabis use disorder and impulsivity, and the second of patients combining cannabis use disorder, absence of insight and non-adherence to treatment. The results also show the moderating role of insight and adherence to treatment on the rate of VB in patients with cannabis use disorder.

**Conclusion:** This study suggests that cannabis use disorder is a significant risk factor for VB amongst early psychosis patients, particularly when combined with either impulsivity, lack of insight and non-adherence to treatment. These results suggest that preventive strategies could be developed on the basis of such patient profiles.

## Introduction

Cannabis use is particularly frequent among psychosis patients (1), especially in early psychosis patients (EPP) (2–7), and is linked with poorer outcomes in schizophrenia (3) as well as EPP (4, 8, 9). A recent paper based on data drawn from the MacArthur study (10) suggests that cannabis use in psychosis patients is also linked to an increased risk of violent behavior (VB) and that persistent cannabis use is a significant predictor of future VB (10). Although the prevalence of cannabis use is well established among violent people, both in the general population (11) and among psychotic patients (6), the link between cannabis use and VB is still a matter of controversy in psychosis (11, 12). Indeed, there is a lack of consensus on the nature of the link and its direction with inconsistencies between studies. In addition, studies have failed to consider potentially confounding dynamic risk factors (10, 13–15). Very few studies explored this issue in EPP (2, 16, 17) while taking into account such potentially confounding factors. In fact, most authors explored the link between VB and substance use in general (2, 6, 18), without focusing specifically on cannabis, despite the high prevalence of cannabis use in EPP patients (17, 19) and the fact that the early phase of psychosis is known to be at high-risk for VB (18, 20–22). Consequently, there is a lack of clear understanding on this important issue.

In addition, establishing the association between the occurrence of a behavior and one risk factor is not sufficient to build a preventive strategy. Indeed, considering possible interplay between different factors is an important part of developing effective interventions (23–25). This means exploring the complex patterns of interactions between factors where the effect of one factor can be neutralized or amplified by the impact of another (26). In order to develop preventive strategies for VB, it is therefore particularly important to analyze the patterns of interaction between dynamic risk factors (features that therapeutic interventions can modify) (12, 21, 27, 28).

Hence, studies on the impact of comorbid substance use (alcohol, cannabis, other substances) and psychosis (29–31) should take into account possible interactions between substance use and main dynamic risk factors of VB identified in the literature, such as positive symptoms (32–34), level of insight (21, 35–37), impulsivity (12, 38, 39), affective instability (40, 41), and treatment adherence (33, 35). Various studies have shown that there are subgroups of patients in which these factors may interact (24, 42–44). Along these lines, Lapworth et al. (45) showed that positive symptoms interact with impulsivity to increase the risk of VB in psychotic patients with comorbid substance use. Other studies on the risk of VB in psychosis revealed the complex interactions between substance use, impulsivity and comorbid antisocial personnality disorder (38, 43, 46), the effect of the interaction between substance use and insight (47, 48) and the links between substance use and poor adherence to treatment (49). Finally, both the lack of clarity about the link between insight and VB, which remains controversial in the literature (35, 50), and the fact that insight has sometimes been considered a risk factor or a protective factor (22, 27), may be due to the way in which insight interacts with other factors.

Identifying such interactions between risk factors and their impact on the occurrence of VB is particularly important in the early phase of psychosis, as it would allow early identification of patients at risk of such behaviors and the development of preventive strategies that could be adapted to subgroups of patient (22, 23, 25, 44).

In this context and considering the high prevalence of cannabis use in the early phase of psychosis, we carried out a study on a sample of early psychosis patients (EPP), in order to explore the impact of cannabis use on the risk of VB, while taking into account (1) potentially confounding factors and (2) interactions with other dynamic factors.

## Methods

### Procedure and participants

Patients were recruited from the Treatment and early Intervention in Psychosis Program (TIPP), a specialized early psychosis program, implemented at the Department of Psychiatry CHUV, in Lausanne, Switzerland (51, 52). Entry criteria to the program are: (i) age 18–35 years; (ii) residence in the catchment area; (iii) meeting threshold criteria for psychosis, as defined by the ‘Psychosis threshold' subscale of the Comprehensive Assessment of At Risk Mental States Scale (CAARMS) (53). Exclusion criteria are (i) antipsychotic medication for more than a total of 6 months, (ii) psychosis related to intoxication or organic brain disease, or (iii) an intelligence quotient < 70. Considering that, like in other similar clinical programs, first treatment for psychosis sometimes occurs only at the time of a second psychotic episode, we consider this cohort is composed of “early psychosis patients” (EPP) rather than strictly first episode psychosis (FEP) patients.

Case managers, who have up to one hundred contacts with patients over the three years of treatment, fill in a specifically designed questionnaire for all patients enrolled in the program. It allows the assessment of demographic characteristics, past medical history, exposure to life events as well as level of symptoms and functioning at the moment of entry in the program. The questionnaire is based on information gathered from patients and their family over the first few weeks of treatment and updated during follow up. Follow-up assessments exploring various aspects of treatment and co-morbidities as well as evolution of functional level are conducted by case managers after 2, 6, 12, 18, 24, 30, and 36 months in treatment. A research psychologist assesses the level of symptoms at each measurement occasion during follow-up. The Research and Ethics Committee of the Faculty of Biology and Medicine of Lausanne University granted access to Treatment and Early Intervention in Psychosis Program clinical data for research purposes.

At the time of this study, 265 patients had been followed-up prospectively over 36 months, and were dichotomised based on the presence or not of VB. The group of Violent Patients had committed physical aggression against people at least once, either before entering into the program and/or during the program, meeting definition of “*serious violence*” i.e., “*as assault causing any degree of injury, any use of a weapon or any sexual assault. The term any was used when the severity of the violence was not specified* (21).”

### Measures

#### Positive symptoms, substance use disorder, insight, impulsivity, affective instability, and treatment adherence

The level of positive symptoms was measured using the positive subscale of the Positive and Negative Syndrome Scale (PANSS) (54). Substance use disorder (SUD) was assessed in 2 ways, first on the basis of DSM-IV criteria (43); secondly being rated dichotomously as “present” or “absent” on the basis of scores from the Case Manager Rating Scale (CMRS) (55). This scale allows the rating of the intensity of substance use on a scale ranging from 1 to 5 (1 being absence and 5 very severe substance use), and ratings corresponding to “absent/light substance use” were scored as absence of SUD, whereas ratings corresponding to “moderate to severe substance use” were scored as presence of SUD. Three types of substances were considered: cannabis, alcohol and other drugs (opioids, cocaine, hallucinogens, and others). The level of insight was rated, using a 3-point scale, as either absent, partial or full. Full insight meant awareness of the illness and the necessity of treatment. Impulsivity was assessed by the addition of 2 PANSS items: “poor impulse control” and “difficulty in delaying gratification,” which correspond to the definition of impulsivity proposed by Moeller et al. (56). Affective instability was rated by the PANSS item “Affective instability,” which assesses unstable, fluctuating, inadequate and/or poorly controlled emotional responses. Treatment adherence was assessed on a 3-point scale of Treatment Adherence Scale [TAS (57)] ranging from 0 to 2: 0 = no adherence; 1 = partial adherence (from 25 to 75% of the time during the evaluation period); 2 = total adherence (from 75 to 100% of the time during the evaluation period).

#### Diagnostic assessment and personality disorders assessment

Diagnosis and comorbid personality disorders were the result of an expert consensus (between psychiatrist and psychologist) and based on the following elements: (1) the disorders reported by treating psychiatrists in all medical documents and at the end of any hospitalization; (2) longitudinal assessment by clinical case managers, after 36 months (58). In this study, the main diagnoses and the comorbid personality disorders were taken into account according to the diagnostic and statistical manual of mental disorders [DSM-IV (59)]. Diagnosis was sub-divided into 5 classes: schizophrenia, schizophreniform disorder, Schizoaffective disorder, major depression with psychotic features, bipolar disorder with psychotic features. We have considered comorbid personality disorders, including antisocial personality disorders.

#### Identification of episodes of violent behavior

Episodes of VB were identified in three distinct ways. First, case managers recorded information regarding the occurrence of violent offenses (Swiss Criminal Code) and VB (such as assault and battery) in a specific chapter of the baseline questionnaire, a reliable method considering the meta-analysis of Winsper et al. (60) that showed good reliability and validity in the self-reporting of serious aggression. Secondly, case managers gathered any additional information through contact with parents, significant others and the forensic psychiatric services (hetero reporting of aggression) over the entire duration of treatment. Finally, episodes of VB occurring during the treatment phase were identified on the basis of the Staff Observation Aggression Scale [SOAS-R scale (61), a structured assessment tool], which lists all critical events related to a VB during hospitalisations (44).

### Statistical analysis

All statistical analyses have been performed using R environment for statistical computing (Packages boot and glm for the logistic regression part and Package rpart for the Classification and Regression Trees and package stats for calculating correlations). As mentioned above, for these analyzes, we considered the dynamic factors assessed at program entry, and conducted the analysis only for patients who committed violent acts during the treatment phase (*N* = 62). Logistic regression was used to assess the direct link between dynamic risk factors [positive symptoms, SUD (cannabis, alcohol and other drugs), insight, impulsivity, affective instability and treatment adherence] and VB committed during the program. We adjusted the model for the main diagnosis and comorbid personality disorder as potential confounding factors. The uncertainty of estimated parameters was assessed using 10,000 bootstrap iterations (62) to ensure the robustness of inferences made based on this model. Bootstrap iterations and fitting steps were performed using “boot” and “glm” functions of R environment for statistical computing, included in “boot” and “stats” libraries respectively.

The Classification and Regression Tree (26) (CART) was used in order to understand the hierarchical effects attributable to the combinations of risk factors on VB, considering that the effects of each of them could be increased or moderated by the presence of others. CARTs are capable of discerning hierarchical associations among a series of explanatory variables and a response variable. Variables with high predictive power appear at the top branches of the tree to form contrasting subgroups of patients. Here, CARTs are constructed using the Recursive Partitioning and Regression Trees algorithm (26) implemented in “rpart” library of Team RC (63). The split rule is based on the Generalized Gini index, which describes the node purity at each node based on each split; the minimum number of observations necessary for a split is fixed at 20. After constructing the tree, pruning can be performed based on the desired complexity of the tree, which is measured by the parameter called Complexity Parameter representing the decrease in relative error rate of the tree if the split at that node is performed. In this analysis, positive symptoms, SUD (cannabis, alcohol and hard drugs), insight, impulsivity, affective instability and treatment adherence were entered as independent variables and VB as the dependent variable.

It is also important to explore the associations between risk factors identified as influential on VB, because a high association among these factors may influence the results and consequently our understanding of their association with the VB as the response. Due to the ordinal nature of these factors, we have used Spearman's correlation on these factors to explore potential associations among them.

## Results

### Descriptive characteristics of the study sample

Violent patients were significantly more likely to be men, with a low level of education and no professional activity. They suffered mainly from schizophrenia and cannabis use disorder. Other detailed characteristics are outlined in Table 1.

**Table 1:** Descriptive characteristics of the study sample of 240 patients.

**Variable**	**%(n) ou M(SD)**	***N* = 240**	**Violent behaviour Physical aggression**		***P*-value**
			**Violent (*N* = 62)**	**Non-violent (*N* = 178)**	
Gender (male) Age at the entry in the program Civil status: single Civil status: married Civil status: divorced Number of years at school Professional activity Student No professional activity	%(n)	67.5 (162) 23.91 (0.31) 85.17 (201) 8.47 (20) 2.97 (7) 9.73 (0.19) 14.17 (34) 20 (48) 65.83 (158)	87 (54) 22.68 (0.56) 85.25 (52) 8.2 (5) 3.28 (2) 8.79 (0.34) 9.68 (6) 11.29 (7) 79.03 (49)	60.7 (108) 24.34 (0.37) 85.14 (149) 8.57 (15) 2.86 (5) 10.04 (0.22) 15.73 (28) 23.03 (41) 61.24 (109)	0.001 0.0154 0.0030 0.0374
Substance Use Disorders	%(n)				
All substances		54.81 (131)	73.77 (45)	48.31 (86)	0.0010
Consumption of substances in the last month (CMRS)					
CMRS alcohol		46.46 (105)	56.67 (34)	42.77 (71)	0.0894
CMRS cannabis		33.04 (75)	60.66 (37)	22.86 (38)	0.0000
CMRS other drugs^a^		3.57 (8)	5 (3)	3.05 (5)	0.7715
Age of onset of cannabis use		16.43 (0.3)	15.29 (0.45)	16.95 (0.37)	0.0052
Impulsivity (2 items)	M(SD)	2.84 (0.1)	3.48 (0.25)	2.64 (0.1)	0.0027
Poor impulse control		1.52 (0.06)	1.88 (0.14)	1.41 (0.06)	0.0040
Difficulty in delaying gratification		1.32 (0.05)	1.61 (0.13)	1.23 (0.05)	0.0090
Absence of Insight	%(n)	34.05 (79)	45.9(28)	29.82 (51)	
Partial insight		47.41 (110)	42.62 (26)	49.12 (84)	
Presence of insight		18.53 (43)	11.48 (7)	21.05 (36)	0.0488
Total PANSS positive	M(SD)	13.28 (0.33)	14.04 (0.72)	13.05 (0.37)	0.2229
Treatment Adherence (TAS)	%(n)	65.18 (146)	55.93 (33)	68.48 (113)	0.1146
Affective instability	M(SD)	1.42 (0.05)	1.63 (0.12)	1.35 (0.05)	0.0334

a *Opioids, cocaine, hallucinogens, volatile solvents*.

### Violent behaviors

Among the 265 patients included in the study, 72 displayed VB that involved a person (27.2%) and 15 (5.7%) a crime against property only; the latter were excluded from the study considering they could be considered neither as control nor as violent patients. Of the 72 patients, 62 have been violent during the program and 10 patients only before entry into the program. The analysis was therefore conducted in a sample of 240 patients (178 Non-Violent Patients and 62 violent patients only during the program).

### Relationship between dynamic factors and VB

Logistic regression model on the dynamic factors, have shown statistically significant association between Cannabis Use Disorder (CUD) and VB [*z*_(3.99)_ = 0.41; *p* = 0.0001]; impulsivity and VB [*z*_(2.15)_ = 0.159; *p* = 0.03], while the links with alcohol and hard drugs use, positive symptoms, insight, affective instability and treatment adherence were not significant. The uncertainties of these parameters were estimated using 10000 bootstrap iterations; the results were not sensitive to the number of bootstrap iterations. When the model was adjusted for the main diagnosis (model 2) and comorbid personality disorder (model 3) no change was observed, and as before CUD and impulsivity remained significantly associated with VB (Table 2).

**Table 2:** Multivariate logistic regression on the dynamic factors and with control of the main diagnosis and personality disorder.

	**Estimate**	**Std. Error**	***z*-value**	**Boot *P*-value**
**MODEL 1**				
Impulsivity	0.343996	0.159434	2.158	0.0163
Insight	−0.207183	0.290284	−0.714	0.2776
PANSS positive	−0.054147	0.049958	−1.084	0.1070
Adherence Scale (TAS)	−0.077752	0.3779	−0.206	0.4271
CMRS alcohol	0.001409	0.399421	0.004	0.4804
CMRS cannabis	1.640373	0.411122	3.99	0.0001
CMRS other drugs^a^	−0.256081	1.233221	−0.208	0.3996
Affective instability	0.288529	0.310281	0.93	0.1929
**MODEL 2**				
Impulsivity	0.35887	0.16904	2.123	0.0178
Insight scale	−0.09836	0.30463	−0.323	0.4335
PANSS positive	−0.05046	0.05205	−0.969	0.1367
Adherence Scale (TAS)	−0.32969	0.41471	−0.795	0.2085
CMRS alcohol	−0.05605	0.41389	−0.135	0.4703
CMRS cannabis	1.84123	0.46383	3.97	0.0000
CMRS other drugs	−0.23099	1.25353	−0.184	0.4166
Affective instability	0.26318	0.3149	0.836	0.2164
Schizophrenia	−0.73398	1.13446	−0.647	0.2414
Schizophreniform/brief	1.12584	0.67183	1.676	0.0529
Schizoaffective disorder	−0.36515	0.96331	−0.379	0.4096
Major depression with psychotic features	1.2252	0.80787	1.517	0.1259
Bipolar disorder	−0.64626	0.72436	−0.892	0.1323
**MODEL 3**				
Impulsivity	0.33156	0.17155	1.933	0.0268
Insight scale	−0.1539	0.30856	−0.499	0.3755
PANSS positive	−0.04952	0.05234	−0.946	0.1388
Adherence Scale (TAS)	−0.33415	0.41995	−0.796	0.1928
CMRS alcohol	−0.01267	0.41727	−0.03	0.4963
CMRS cannabis	1.68646	0.47304	3.565	0.0002
CMRS other drugs	−0.28079	1.22652	−0.229	0.4058
Affective instability	0.31059	0.317	0.98	0.1881
Schizophrenia	−0.85236	1.14064	−0.747	0.2115
Schizophreniform/bref	1.11242	0.66575	1.671	0.0527
Schizoaffective disorder	−0.90105	1.10558	−0.815	0.2813
Major depression with psychotic features	1.17661	0.80819	1.456	0.1323
Bipolar disorder	−1.03081	0.82903	−1.243	0.0634
Comorbid Personality disorder	1.4752	0.97793	1.508	0.0823

a *Opioids, cocaine, hallucinogens, volatile solvents. The gray color shows the significant P-value*.

### Combined hierarchical effect of factors on the rate of VB

**Figure 1:**
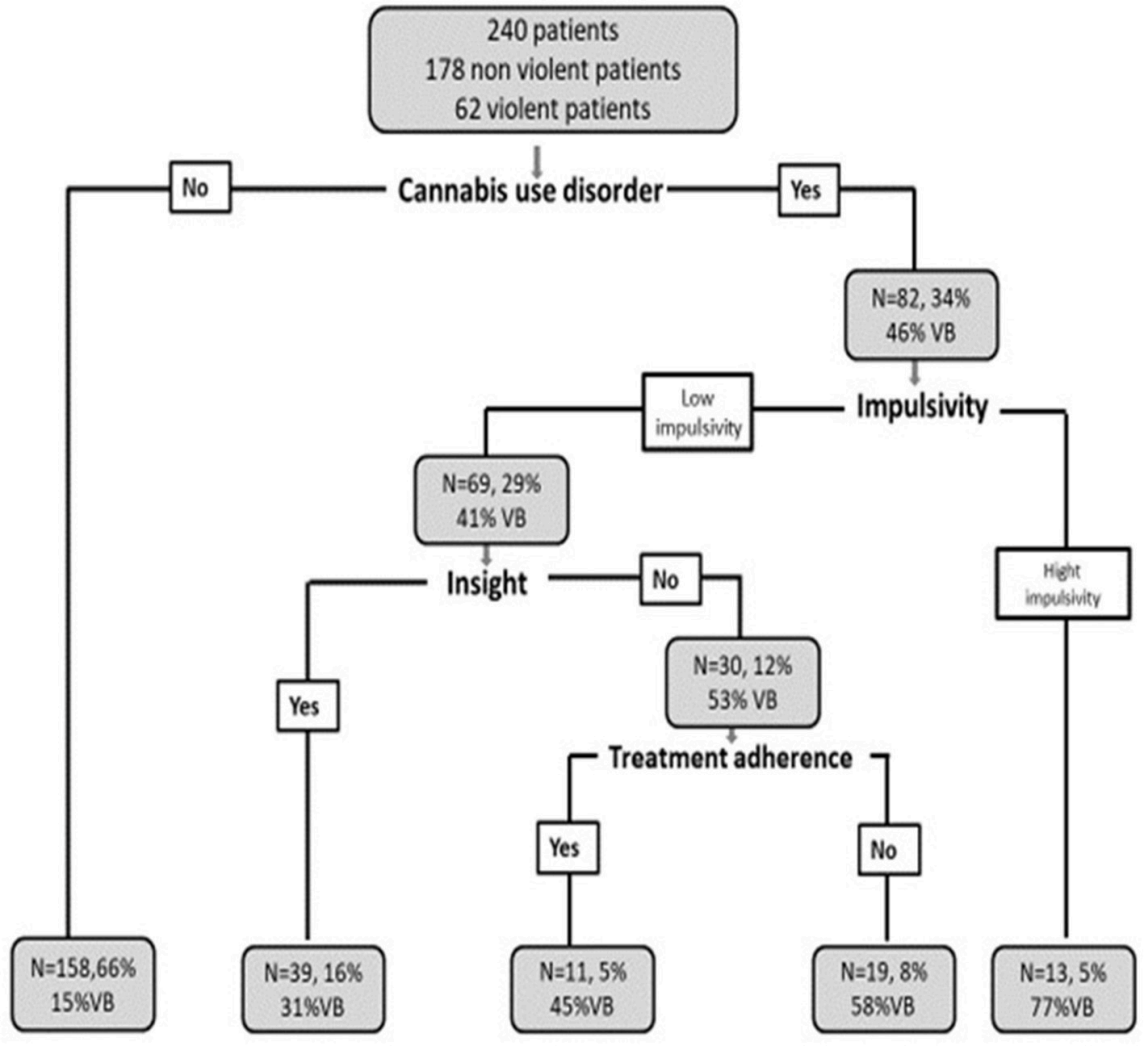
Combined hierarchical effect of factors on the rate of VB. *N* = Total number of patients; % = Percentage of patients in the total sample; % of VB, Percentage of VB in the subgroup studied.

The CART analysis (see Figure 1) revealed the role played by dynamic factors by exploring their hierarchical mutual effects and patient subgroups. To hierarchically describe the importance of factors influencing membership in subgroup, the factors mentioned are decisive for distinguishing subgroups and each cell describes the number of patients, the percentage on the sample and the percentage of VP in each subgroup.

CART analysis has identified four main factors presented in decreasing order of importance. First factor: CUD; second: impulsivity; third: insight and fourth factor: treatment adherence. The other studied factors did not appear in the results of this analysis.

Subgroups of patients: CUD plays an important role in VB (first factor that dichotomized the tree). In the subgroup without CUD, only 15% of patients (*N* = 24/158) displayed VB, and 85% of them did not. In the CUD subgroup, 46% of patients (*N* = 38/82) displayed VB. In this subgroup, Impulsivity dichotomized the tree in two: in a first little subgroup (*N* = 13), composed of patients with CUD, presence of impulsivity (higher than 4.5) led to a 77% of VB in this subgroup (*N* = 10/13). In the second (*N* = 69), when such patients were cannabis user, had low level of impulsivity (under 4.5) but had lack of insight (lower than 0.5), the rate of VB raised to 53% (*N* = 16/30), while it fell to 30.7% (*N* = 12/39) in presence of insight. 69% (*N* = 27/39) of patients in the subgroup with insight did not display VB. Among patients with CUD, lack of insight and lack of treatment adherence were linked to a 58% rate of VB (*N* = 11/19), while 45% of such patients (*N* = 5/11), had displayed VB if they had treatment adherence.

In order to understand the relationships between factors that emerged in CART analysis, we explored correlations between factors. We observed that (a) CUD is not correlated to impulsivity significantly, (b) CUD is negatively correlated both with insight (*p* = 0.002; rho −0.206), and treatment adherence (*p* = 0.000; rho −0.232); (c) lack of insight and lack of treatment adherence were not linked together. Relatively small correlation coefficients observed here will not impose any multicollinearity between factors in CART analysis.

## Discussion

To our knowledge, this is the first study attempting to explore the interaction between CUD, various dynamic risk factors, and the risk of VB in the early phase of psychosis. Our main findings are the following. First, CUD is the strongest risk factor of VB. Second, impulsivity seems to be an important risk for VB. Third, the exploration of the interactions between risk factors suggest the presence of two main patient profiles, who display a combination of CUD and dynamic risk factors, which lead to an increased rate of VB. Fourth, insight and treatment adherence play an indirect role, moderating the rate of VB in EPP who use cannabis.

### Cannabis use disorder and impulsivity

In our EPP cohort, classically composed of two thirds of males aged on average 24 at treatment entry, 33% of patients use cannabis, which is in line with data provided by other cohorts (3). Interestingly, the prevalence of CUD was 61% in patients who displayed VB, while it was only of 23% in patients who did not, suggesting a link between these two factors. After controlling for the impact of alcohol abuse, other forms of substance abuse, dynamic risk factors, diagnosis and personality disorder comorbidity, our results confirm previous data (5) showing that CUD is a significant risk factor for the occurrence of VB (51), and show this specifically in an early psychosis population. Previous research in similar populations showed that substance abuse in general is a risk for VB (17, 18, 22, 27). Establishing that CUD plays a specific role in this matter is important considering its high prevalence in EPP and it's early onset at age 15 in violent patients (6, 7, 64, 65), and the fact that it can be influenced by treatment, especially in young patients (7, 66). Specialized early intervention programs should therefore include specific interventions to treat CUD in order to prevent not only poorer outcome (2, 5) but also the occurrence and persistence of VB (10).

The study of the nature of the links between impulsivity and VB has produced contradicting results, some studies supporting a direct relationship with VB, while others suggested this link would be indirect (38). Although this link in psychosis patients is a matter of controversy (12, 46, 67–69), our results are in support of a positive association between impulsivity and VB. However, since impulsivity is a multifaceted construct (70, 71), this result must be confirmed in future studies applying a multi-dimensional scale to assess this dimension. This would allow to examine whether certain specific dimensions of impulsivity (in particular lack of control and difficulty in delaying satisfaction) rather than a global trait of impulsivity are related to VB.

### Two patterns of interaction of factors

The use of CART analysis allowed us to identify patient subgroups where various risk factors seem to interact and to classify them according to a hierarchical order of importance. This information may provide useful information for the development of treatment strategies.

#### Subgroup 1: cannabis use disorder and impulsivity

The first violent patients subgroup, composed of patients displaying CUD and impulsivity had the highest rate of VB, and the combination of these two factors explained 77% of the occurrences of VB in this subgroup. It is likely that the combination of a tendency to react impulsively combined with the disinhibiting effect of CUD leads to reactive and unplanned violent acts (42, 72), in response to everyday situations. In addition, impulsivity can lead to substance abuse (73, 74), which in turn can increase impulsivity (69), leading to a pernicious vicious circle. Finally, from a neurodevelopmental point of view, abuse of illicit substances, which often starts in adolescence (our results showed that the age of onset of cannabis use in violent patients was 15 years versus 17 years in non-violent patients), could have a deleterious impact on the developing brain, which is particularly sensitive to the neurotoxic effect of such compounds (69, 75, 76). Indeed, the results of a recent meta-analysis of imaging studies exploring the link between cannabis use and impulsivity suggests cannabis has an impact both on the structure and on the function of the brain, notably at the level of the prefrontal cortex which is specifically involved in the control of behavior (77).

#### Subgroup 2: cannabis use disorder, lack of insight and lack of treatment adherence

The second high-risk subgroup of VB was composed of cannabis users who simultaneously exhibited an absence of insight and non-adherence to medication. While the multivariate logistic regression suggested insight has no direct impact on VB, the results of CART analysis revealed that insight indeed had an influence on VB, but only in the subgroup of cannabis user, its absence being linked to 53% of VB in this subgroup. These results suggest that insight has an indirect impact on VB, and that it plays a moderating role in this matter, which may explain why the literature has been inconsistent on this issue in the past (22, 35, 50). In line with previous findings on protective factors against VB (13, 67), we found that the presence of insight and adherence to treatment decreases the likelihood of VB, in EPP, and specifically here, in patients who use cannabis.

In this second subgroup, in line with Swartz study's (78), our results show that combination of substance abuse, lack of insight and treatment adherence increases the risk of VB (58% of VB). Our analysis allowed the exploration of the interaction between the three factors at play in this subgroup. The literature has identified that these factors can be mutually reinforcing, which may explain the potentiation of their effects to increase the VB rate in this subgroup. To our knowledge, few studies directly addressed the effect of cannabis use on insight level (or vice versa). Some studies showed that cannabis use is linked to lack of insight (47, 48). Whereas, others studies revealed that cannabis use has an effect on both treatment resistance (4, 21) and non-recognition of illness, in that patients may attribute symptoms to the effects of cannabis. In addition, some authors have shown that treatment non-adherence and substance abuse can reinforce each other to the extent that cognitive difficulty associated with drug use can impede compliance. Further, Ekinci (79) mentions that the combination of lack of insight and lack of treatment adherence may increase VB, by impeding the recognition of psychotic symptoms. Based on the above-mentioned work (79), we can hypothesize that difficulty induced by the combination of these three factors could contribute to increase cognitive distortions and difficulties in social interactions which in turn could lead to aggressive reactions against others (44, 50). If this were to be proven in future research, the complex intrication of these factors would justify the development of an integrated treatment strategy targeting these three dimensions simultaneously (48).

## Limitations

Our study suffers from various limitations. First, the small sample size excludes the generalization of these results. Second, impulsivity was measured on the basis of 2 PANSS items rather than on the basis of a specialized scale. Third, assessment of positive symptoms was not always simultaneous to the occurrence of VB. Fourth, although we used different methods in order to assess the occurrence of VB (self-reporting by patients, hetero-reporting by relatives, information stemming from the forensic services and assessment by a standardized instrument), occurrence of VB prior to entry into the program might have been under evaluated, due to absence of access to police and criminal records. Fifth, although the analysis was conducted on the basis of some of the main potential dynamic risk factors for violent behavior, other potential dynamic risk factors may be considered in future studies. In addition, as mentioned above, assessment of personality disorder was the result of an expert consensus but was not based on a specific assessment, and it is therefore not excluded that some aspects of this diagnosis may have been missed. Finally, the result of our analysis is mainly correlational and we can't exclude that “inherently” violent patients may have started to use cannabis in an attempt to experience a calming effect.

## Conclusion

Considering CUD is highly prevalent amongst patients with psychosis (1) and even more so amongst EPP (2, 3), our results suggest it should be the target of early intervention, especially when combined with either impulsivity or lack of insight considering the observed association between CUD and violent behavior. Our results also suggest more research is needed regarding protective factors that may actually decrease risk of VB in patients despite their cannabis use; this would allow the development of interventions that would focus on resources of patients and their environment, which may, as indicated by a few studies (80–82), facilitate patients' engagement in such treatments (83–86).

## Author contributions

VM wrote the manuscript with contribution of PC and contributions from all authors. MG performed the statistical analyses. All authors contributed to and have approved the final manuscript.

### Conflict of interest statement

The authors declare that the research was conducted in the absence of any commercial or financial relationships that could be construed as a potential conflict of interest.
